# Prevalence of neurological conditions across the continuum of care based on interRAI assessments

**DOI:** 10.1186/1472-6963-14-29

**Published:** 2014-01-22

**Authors:** Oana Danila, John P Hirdes, Colleen J Maxwell, Ruth Ann Marrie, Scott Patten, Tamara Pringsheim, Nathalie Jetté

**Affiliations:** 1 School of Public Health and Health Systems, University of Waterloo, Waterloo, Canada; 2 School of Pharmacy, University of Waterloo, Waterloo, Canada; 3 Departments of Internal Medicine and Community Health Sciences, University of Manitoba, Winnipeg, Canada; 4 Department of Psychiatry, University of Calgary, Calgary, Canada; 5 Department of Community Health Sciences, University of Calgary; 6 Mathison Centre for Research and Education in Mental Health, University of Calgary, Calgary, Canada; 7 Department of Clinical Neurosciences, University of Calgary, Calgary, Canada; 8 Department of Pediatrics, University of Calgary, Calgary, Canada; 9 Hotchkiss Brain Institute and Institute for Public Health, University of Calgary, Calgary, Canada

**Keywords:** Nursing homes, Home care, Psychiatry, Epidemiology, interRAI

## Abstract

**Background:**

Although multiple studies have estimated the prevalence of neurological conditions in the general Canadian population, limited research exists regarding the proportion affected with these conditions in non-acute health care settings in Canada. Data from standardized clinical assessments based on the interRAI suite of instruments were used to estimate the prevalence of eight neurological conditions across the continuum of care including Alzheimer’s disease, Parkinson’s disease, epilepsy, traumatic brain injury, multiple sclerosis, cerebral palsy, Huntington’s disease, and amyotrophic lateral sclerosis.

**Methods:**

Cohorts of individuals receiving care in nursing homes (N=103,820), home care (N=91,021), complex continuing care (N=10,581), and psychiatric hospitals (N=23,119) in Canada were drawn based on their most recent interRAI assessment within each sector for a six-month period in 2010. These data were linked to the Discharge Abstract Database and National Ambulatory Care Reporting System data sets to develop five different case definition scenarios for estimating prevalence.

**Results:**

The conditions with the highest estimated prevalences in these care settings in Canada were Alzheimer’s disease and related dementias, Parkinson’s disease, epilepsy, and traumatic brain injury. However, there were notable cross-sector differences in the prevalence of each condition, and regional variations. Prevalence estimates based on acute hospital administrative data alone were substantially lower for all conditions evaluated.

**Conclusions:**

The proportion of persons with neurological conditions in non-acute health care settings in Canada is substantially higher than is generally reported for the general population. It is essential for these care settings to have the expertise and resources to respond effectively to the strengths, preferences, and needs of the growing population of persons with neurological conditions. The use of hospital or emergency department records alone is likely to substantially underestimate the true prevalence of neurological conditions across the continuum of care. However, interRAI assessment records provide a helpful source of information for obtaining these estimates in nursing home, home care, and mental health settings.

## Background

Estimates of the prevalence of diseases are important to various stakeholders, as they can be used to plan service needs, allocate health care resources, prioritize research expenditures, and raise awareness about the impact of specific conditions. Geographic variations in prevalence may provide insights into risk (or protective) factors associated with a given condition. Also, differences in the prevalence of conditions across the continuum of health care settings provide information about how health services might be restructured to meet the needs of future populations. This is particularly important for neurological conditions as many are chronic, progressive and lifelong conditions which can as a result have a substantial impact on health care utilization [1].

Neurological conditions account for about 6% of the global burden of disease, and their prevalence is expected to continue to rise with the aging of population [2]. In Canada, previous research has focused mainly on estimating the prevalence of the most common neurological conditions such as dementia, multiple sclerosis, epilepsy, and Parkinson’s disease [3-8]. A paucity of estimates exists regarding the prevalence of less common conditions including Huntington’s disease, cerebral palsy, dystonia, and tic disorders [9-12]. From the perspective of policy-makers and service providers, a common limitation of most research on neurological conditions is the tendency to only consider prevalence in the general population rather than within specific care settings. Further, studies of persons in settings like nursing homes have tended to rely on survey data, which may be affected by sampling and non-response biases [3].

The interRAI family of assessment instruments can provide valuable information about the prevalence of neurological conditions and their impact on health and well-being [13-15]. The large scale implementation of interRAI instruments across the continuum of care in Canada offers a new opportunity to estimate the prevalence of a broad range of neurological conditions in home care, nursing home, and hospital settings [16-22]. The present study is the first to use interRAI data holdings linked to other health records to estimate the prevalence of neurological conditions across the continuum of care as part of the Innovations in Data, Evidence and Applications for Persons with Neurological Conditions project (ideasPNC), funded by the Public Health Agency of Canada.

The aim of this study was to examine the prevalence of neurological conditions in home care, nursing homes, complex continuing care, and psychiatric hospitals/units. The neurological conditions of interest included Alzheimer’s disease and related dementias (ADRD), Parkinson’s disease (PD), epilepsy, traumatic brain injury (TBI), multiple sclerosis (MS), cerebral palsy (CP), Huntington’s disease (HD), and amyotrophic lateral sclerosis (ALS).

## Methods

### Data sets used

The data sets used for case definitions and their corresponding standard data collection forms are all managed by the Canadian Institute for Health Information (CIHI). The clinically based data sets derived from interRAI assessment records include the Home Care Reporting System (HCRS) – RAI-HC, Continuing Care Reporting System (CCRS) – RAI 2.0, and Ontario Mental Health Reporting System (OMHRS) – RAI-MH. The administrative CIHI data sets used were the Discharge Abstract Database (DAD) – hospital visit abstract, and the National Ambulatory Care Reporting System (NACRS) – emergency department visit abstract.

Following data submission and quality checks, CIHI de-identifies the individual level observations and then re-assigns to each of them a new unique identifier, designed to ensure that the true identity of any given person cannot be recovered. However, this unique identifier can be used to link all data sets mentioned above. Therefore, it is possible to follow a person over time not only within one sector, but throughout all care settings, including acute and ambulatory care. The linked data sets were provided by CIHI based on a data sharing agreement as part of the ideasPNC project. The ideasPNC project received ethics approval from the Office of Research Ethics, University of Waterloo (project #17045).

#### Home Care Reporting System (HCRS)

The HCRS data set contains demographic, clinical, functional and resource utilization information about persons receiving publicly funded home care programs in Canada. The standard form used for assessment is the RAI-HC form. RAI-HC was fully implemented in Ontario in 2006, in Nova Scotia in 2004, and in Yukon in 2010. Persons receiving home care are assessed at admission, discharge, and also annually if services are received for more than a year. Ontario clients are assessed bi-annually.

There are 864,955 records with non-missing unique client identifier and date of assessment, collected from 362,698 persons between 2001 and 2011. The total number of persons receiving home care stabilizes around 2008 for all provinces/territories, with the exception of Nova Scotia as agencies in this province have not submitted RAI-HC assessments to CIHI since the first quarter of 2010 (see Additional file 1: Table S1 for more details).

The RAI-HC instruments include a record of the person’s current diagnoses completed by a trained assessor using all sources of information available including previous medical records. A “pick list” includes four neurological conditions of interest for this study (i.e., ADRD, TBI, MS, and PD). Although there are items designated for recording “International Classification of Diseases” version 10, Canada (ICD-10-CA) codes [23], the de-identified HCRS data set provided by CIHI does not include those open-ended items. Hence, for persons receiving home care, prevalence estimates are obtained only for diagnoses specifically listed on the RAI-HC form, namely ADRD, TBI, MS, and PD (see Additional file 1: Table S6 for details).

#### Continuing Care Reporting System (CCRS)

The CCRS data set includes standardized measures for residents of long-term care facilities (i.e., nursing homes), and hospital-based continuing care facilities (i.e., complex care or chronic care hospitals or units). The CCRS contains longitudinal demographic, clinical, functional, and resource utilization information about persons in nursing homes and complex care hospitals, based on the RAI 2.0 assessment. For long-term care residents, RAI 2.0 was mandated in British Columbia, Winnipeg Regional Heath Authority (WRHA) in Manitoba, Ontario, and Yukon, and it was fully implemented in these provinces/territories by 2010. In Nova Scotia, the implementation is optional for nursing homes, and in Newfoundland and Labrador, implementation was underway but not completed at the time the study data set was constructed. For complex-continuing care clients, RAI 2.0 was fully implemented in Ontario in 1996. Also, the instrument was implemented in one chronic-care hospital in WRHA by 2010. Residents in all participating provinces/territories are assessed at admission, quarterly with a shorter version of RAI 2.0, and annually using the full version of the instrument.

The CCRS data set contains 1,577,614 records with non-missing unique identifiers and dates of assessment, collected from 299,032 persons between 2003 and 2011. For most provinces/territories, the number of persons assessed every year increases over time and then stabilizes around 2009. This can be explained by the gradual implementation of the RAI 2.0 instrument across Canada (see Additional file 1: Table S2 for more details).

RAI 2.0 assessments contain information related to diagnoses, either as part of a pick-list for various conditions or as ICD-10-CA codes. See Additional file 1: Tables S7–S8 for more details related to the diagnosis of neurological conditions.

#### Ontario Mental Health Reporting System (OMHRS)

The OMHRS data set contains information from the RAI-MH assessments completed on individuals admitted to inpatient mental health beds in general and specialty facilities in Ontario. The RAI-MH instrument includes information related to physical health, social support, and service utilization similar to RAI 2.0 and RAI-HC, but also includes mental-health specific information. Persons receiving inpatient mental-health care are assessed at admission, discharge, and at three-month intervals if the length of stay is three months or greater. The instrument was fully implemented in Ontario in 2006. There are 470,586 assessments recorded in the OMHRS data set since 2005 for 131,948 persons (see Additional file 1: Table S3 for more details).

The RAI-MH instrument contains information regarding medical diagnosis. The given pick-list does not include any neurological condition of interest, but it does provide fields for recording ICD-10-CA codes. Also, the psychiatric diagnostic section provides two additional items that are relevant for identifying an ADRD case: “delirium, dementia and amnesic and other cognitive disorders” in the provisional psychiatric diagnostic section, and two items in the “Psychiatric Diagnosis” – Axis I and II – where DSM-IV codes can be recorded. The latter items are required for completion at discharge, but are optional in the admission assessment.

#### Discharge Abstract Database (DAD)

The DAD is a national database that contains demographic, administrative, and clinical data on inpatient hospital discharges. The input document is the discharge abstract, where relevant discharge information from the chart is entered. Data elements that refer to related concepts are grouped together, and diagnostic information is included in the Abstracting Information Group 10 – Diagnosis. At least one diagnosis has to be recorded in each abstract, with the “most responsible diagnosis” recorded as the first one for each patient. In total, up to 25 diagnoses can be recorded using the ICD-10-CA codes.

Additional file 1: Table S4 shows the number of persons with at least one interRAI assessment in either the CCRS, HCRS, or OMHRS data sets, who had hospital visits, by year and by province/territory.

#### National Ambulatory Care Reporting System (NACRS)

The NACRS contains data on hospital-based and community-based ambulatory care. Data collected during a person’s visit to the emergency department (ED) include demographic, clinical, administrative, financial, and service information. Diagnostic information is recorded using ICD-10-CA codes, and includes the most responsible diagnosis for the ED visit, and up to nine additional diagnoses.

Additional file 1: Table S5 shows the number of persons with at least one interRAI assessment in either the CCRS, HCRS, or OMHRS data sets, who had ED visits, by year and by province/territory.

### Cohort definition

To estimate the period prevalence of the eight neurological conditions of interest, a specific cohort for each care setting was selected as follows.

First, a time frame (called the “index interval”) was specified to be January 1st to July 1st, 2010. This index interval was common for all care settings. For each care setting, all persons who had at least one assessment during the index interval were included in the cohort. As persons can have multiple assessments during the index interval, the most recent assessment, called the “index” assessment, was selected. The date corresponding to the index assessment became the “index date”.

**Figure 1:**
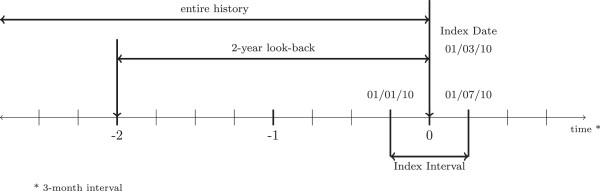
Time frame for one person.

The prevalence for a neurological condition within these specific cohorts was estimated based on the proportion of cases that were identified using five different scenarios. These scenarios use diagnostic information from different sources, including the index assessment, over different periods of time (e.g., two-year look-back window or entire history prior to a person’s index date), and will be described in more detail later on. Figure 1 shows the time line for one person, where the starting point for case definition is the index date.

Data related to the HC and MH cohorts are full-population inventories at the provincial/territorial level, as the implementation of RAI-HC and RAI-MH was complete in the corresponding provinces at the beginning of the index interval (i.e., January 1st to July 1st, 2010). That is, the HC cohort includes all persons receiving long-term home care in Ontario and Yukon. The MH cohort includes all adults receiving inpatient psychiatric care in Ontario, during the index interval. However, as Nova Scotia only reported HC data for the first quarter of 2010, the corresponding cohort includes all persons receiving long-term home care during the first quarter of 2010.

Data related to the LTC cohorts from British Columbia, Ontario, and Yukon are full-population inventories at provincial/territorial level. For Manitoba, the data are full-population inventories at the Winnipeg Region level. For Nova Scotia and Newfoundland and Labrador, the cohorts do not include all existing LTC facilities. Data related to these cohorts are full-population inventories at the facility level, but represent a convenience sample at the provincial level.

The data corresponding to the CCC cohort in Ontario are full-population inventories at provincial level. There is also a small cohort of CCC patients from one reporting hospital in Manitoba.

### Extracting relevant diagnostic information from all sources

For each care setting, a data set containing all information from the index assessments (i.e., information from most recent assessment of persons in a cohort), called the index data set, was created. Within each index data set, binary variables corresponding to whether a person had a specific neurological condition recorded in the index assessment were assigned, using the diagnostic pick-list variables and the ICD-10-CA codes as described in the Additional file 1.

Next, for each person in a cohort, information related to diagnostic history from all interRAI sources, and from DAD and NACRS data sets was extracted. The following example illustrates the procedure for extracting historical diagnostic information from the DAD data set, for a person included in the LTC cohort. The same steps were followed for all the other sources (e.g., NACRS, HCRS), for all persons in the LTC cohort.

First, all DAD records corresponding to this person were selected. Then, two summary binary variables for each neurological condition were created, using the ICD-10-CA codes records. The first one corresponded to whether the person had a record for a specific neurological condition in any of the available records within the DAD data set at any time prior to the index date. The second variable corresponded to whether the person had a record for a specific neurological condition in the DAD data set within two years prior to the index date.

These historical variables, along with the ones extracted from the other sources (e.g., NACRS, HCRS), were appended to the index data set and a new “augmented” index data set was created. Cases of a specific condition were identified based on the information available in this data set and using different rules for case definition as discussed in the next section.

SAS version 9.2 (SAS Institute, Cary, NC) was used for all data extraction and analysis. The SAS integrated Structured Query Language (SQL) module (i.e., PROC SQL) was used extensively for linking the data sets and extracting the historical diagnostic information.

### Scenarios for case definition

Five scenarios for case definition that are common for all neurological conditions were defined, with the first one being the least restrictive in terms of the number of sources used and length of look-back window, to the last two scenarios being the most restrictive. Table 1 shows the information used for case definition, for each scenario.

**Table 1:** Scenarios for case definition of neurological conditions

**Scenario**	**Sources condition**	**Look-back**
	**is present**	**window**
1	index assessment or	index date
	CCRS or HCRS or OMHRS or DAD or NACRS	entire history
2	index assessment or	index date
	CCRS or HCRS or OMHRS or DAD or NACRS	2 years
3	index assessment or	index date
	DAD or NACRS	2 years
4	index assessment	index date
5	DAD or NACRS	2 years

For scenarios 1 – 4, the prevalence estimate for a neurological condition in a specific care setting was obtained as the number of cases identified by the specific scenario over the size of the corresponding cohort.

For scenario 5, the denominator population included only persons who had at least one hospital or ED visit in the last two years.

Given that the timing of implementation of interRAI instruments differs across provinces/territories and also that, for some provinces/territories, DAD and NACRS records were not available (e.g., Yukon territory, see Additional file 1: Tables S4–S5), the prevalence estimates were calculated by province/territory and not pooled nationally. Only prevalence estimates given by scenario 4 (i.e., based on the index interRAI assessment) can be compared across provinces, because that scenario is the only one unaffected by differing availability of historical records.

For all five scenarios, point estimates are provided for the six-month period prevalence (January 1st - July 1st, 2010) and the associated exact 95% confidence intervals (CI), for all neurological conditions within each of the four care settings. Also, *χ*^2^ tests for homogeneity of prevalence given by scenario 4 across provinces/territories were conducted. SAS version 9.2 (SAS Inc., Cary, NC) was used to obtain all these results.

Using historical diagnostic invformation from the interRAI data sets, and DAD and NACRS allows for more cases of a certain neurological condition to be ascertained. It is important to remember that, in the majority of cases, the neurological conditions of interest are incurable conditions; therefore, once a person is correctly diagnosed with a condition, the diagnosis should appear in all future records. Therefore, using historical data can help reduce the bias of the prevalence estimator due to failure to record the diagnosis. However, using historical diagnostic data also poses the risk of including cases where a person is wrongly diagnosed with a certain condition. It is possible that further evaluation indicates that the person does not have the condition, and therefore the condition is not included in future records. In this case, defining the client as a case based on historical records can inflate the prevalence.

It is acknowledged that none of the scenarios presented represents a gold standard for case definition, as errors can occur in both historical records and most recent ones. The main reason for combining different sources of information is to obtain lower and upper bounds for the prevalence estimate of a condition.

## Results

For each care setting, the number of persons in the cohort (*N*), the number of persons with at least one hospital or ED visit within last 2 years (*n*), the mean and standard deviation for age in years, and the percentage of females, by province/territory, are given in Table 2.

**Table 2:** Demographic characteristics of persons in cohorts, by sector and province/territory – January 1st to July 1st, 2010

**Sector &**	**N**^***a***^	**n**^***b***^	**Age**		**Sex**^***c***^
**province/territory**			**Mean**^***d***^	**STD**	**(%)**
**Home care**					
Nova Scotia	4,561	3,058	77.0	14.2	NA^*e*^
Ontario	86,378	72,927	77.7	13.4	65
Yukon	82	53	74.7	13.2	57
**Long term care**					
British Columbia	16,307	8,405	83.8	10.3	68
Manitoba	5,714	2,534	84.7	9.5	73
Newfound. & Labrador	382	194	78.2	12.8	64
Nova Scotia	625	0	86.2	8.5	59
Ontario	80,663	54,425	83.2	10.2	70
Yukon	129	109	77.6	13.7	56
**Complex cont. care**					
Manitoba	138	101	71.5	15.4	57
Ontario	10,443	9,578	76.4	14.1	55
**Mental health care**					
Ontario	23,119	21,602	43.1	16.9	48

^*a*^Total number of persons in the cohort.

^*b*^Number of persons with at least one hospital or ED visit in last 2 years.

^*c*^Percentage of females.

^*d*^Mean and standard deviation of age for all N persons in the cohort.

^*e*^Gender information not available.

The subset of persons selected as the cohort for the present analyses for the HC sector included 91,021 persons. Sixty eight percent of these persons had previous records within HCRS, 10% had a CCRS history, whereas 72% had a DAD, and 88% a NACRS history (results not shown, but available on request).

The subset of persons selected as the cohort corresponding to the LTC sector contained 103,820 persons. Overall, 91% of the LTC clients had a history within the CCRS data set, 35% had a HCRS history, 4% had an OMHRS history, 74% had a DAD history, and 73% a NACRS history (results not shown, but available on request).

The cohort corresponding to CCC hospitals in Ontario included 10,443 patients. Forty six percent of these patients had a CCRS history, whereas 45% had a HCRS history, 94% a DAD, and 97% a NACRS history (results not shown, but available on request). There were also 138 patients from one chronic care hospital in Manitoba.

The MH cohort in Ontario included 23,119 persons, and of these cases, 84% had an OMHRS history. Additionally, 44% had a DAD and 96% had a NACRS history. There are very few in the cohort who had a CCRS or a HCRS history (results not shown, but available on request).

### Prevalence estimates among persons receiving home care

The prevalence estimates for four neurological conditions (i.e., ADRD, PD, TBI, and MS) among the home care clients from Ontario, Nova Scotia, and Yukon, and their associated 95% exact confidence intervals are given in Table 3. For Ontario and Yukon, the prevalence estimates are six-month estimates (i.e., first six months of 2010). However, as Nova Scotia only reported HC data for the first quarter of 2010, the corresponding prevalence estimates are three-month period estimates.

**Table 3:** Estimates of the prevalence of neurological conditions by province/territory, among home care clients assessed between January 1st and July 1st, 2010

	**Province/territory**					
**Condition**^***a***^	**Nova Scotia**		**Ontario**		**Yukon**	
**Scenario**^***b***^	**(%)**	**(CI)**^***c***^	**(%)**	**(CI)**	**(%)**	**(CI)**
**ADRD**						
1	25.0	(23.7,26.2)	25.6	(25.3,25.9)	8.5	(3.5,16.8)
2	24.2	(23.0,25.5)	24.8	(24.5,25.1)	8.5	(3.5,16.8)
3	24.1	(22.8,25.3)	24.0	(23.7,24.3)	8.5	(3.5,16.8)
4 ^*d*^	22.3	(21.1,23.5)	21.6	(21.3,21.9)	8.5	(3.5,16.8)
5	11.7	(10.6,12.9)	10.3	(10.0,10.5)	3.8	(0.5,13.0)
**PD**						
1	4.3	(3.8,5.0)	5.2	(5.0,5.3)	1.2	(0.0,6.6)
2	4.1	(3.6,4.8)	5.0	(4.8,5.1)	1.2	(0.0,6.6)
3	4.1	(3.5,4.7)	4.8	(4.7,4.9)	1.2	(0.0,6.6)
4	3.7	(3.2,4.3)	4.4	(4.3,4.5)	1.2	(0.0,6.6)
5	2.5	(2.0,3.1)	2.5	(2.4,2.6)	0.0	(0.0,0.0)
**TBI**						
1	2.5	(2.0,3.0)	3.2	(3.1,3.3)	7.3	(2.7,15.2)
2	2.1	(1.7,2.6)	2.5	(2.4,2.6)	7.3	(2.7,15.2)
3	2.0	(1.6,2.5)	2.3	(2.2,2.4)	7.3	(2.7,15.2)
4	1.6	(1.3,2.0)	1.2	(1.2,1.3)	7.3	(2.7,15.2)
5	0.9	(0.6,1.3)	1.4	(1.4,1.5)	0.0	(0.0,0.0)
**MS**						
1	2.2	(1.8,2.7)	1.8	(1.7,1.9)	0.0	(0.0,0.0)
2	2.1	(1.7,2.6)	1.8	(1.7,1.8)	0.0	(0.0,0.0)
3	2.0	(1.6,2.5)	1.7	(1.6,1.8)	0.0	(0.0,0.0)
4	2.0	(1.6,2.4)	1.6	(1.5,1.7)	0.0	(0.0,0.0)
5	1.4	(1.0,1.9)	1.0	(0.9,1.1)	0.0	(0.0,0.0)

^*a*^ADRD = Alzheimer’s Disease and Related Dementias, PD = Parkinson’s Disease, TBI = Traumatic Brain Injury, MS = Multiple Sclerosis.

^*b*^See Table 1 for description of each of the five case definition scenarios.

^*c*^Exact 95% confidence interval.

^*d*^Only estimates by Scenario 4 are comparable across provinces.

As expected, the prevalence estimates decreased as the case definition used fewer data sources and shorter time spans for historical data. For example, when comparing the prevalence estimates of ADRD based on the RAI-HC assessment only (i.e., scenario 4) to the ones given by scenario 1, the scenario 4 estimates were lower by 4% in Ontario and by around 3% in Nova Scotia.

ADRD had the highest prevalence estimates among the four neurological conditions. In Nova Scotia, the prevalence estimates ranged from 11.7*%* to 25%, whereas in Ontario they ranged from 10.3*%* to 25.6*%*. In Yukon, the ADRD prevalence estimate was 8.5*%* for all scenarios except for scenario 5.

Note that the prevalence given by scenario 5 (i.e., the scenario where only hospital and ED visits records in the last two years were used for case definition) was the lowest compared to the other scenarios, with estimates ranging from 3.8*%* to 11.7*%* across provinces/territories. Therefore, although very useful as an additional source of diagnostic information, the hospital/ED records did not represent a sensitive source for identifying cases when used on their own. Based on this observation, scenario 5 is no longer considered in further discussion about prevalence estimates (results are still shown in the corresponding tables for information only).

The *χ*^2^ test for homogeneity suggested that the prevalence for ADRD differed significantly across provinces/territories (*p*−*value*=0.009).

The prevalence estimates for PD were generally less than 5% for Nova Scotia and Ontario, whereas in Yukon they were 1.2*%* for all scenarios. The PD prevalence given by scenario 4 differed significantly across provinces/territories (*p*−*value*=0.04).

TBI prevalence estimates ranged from 1.2*%* to 3.2*%* in Ontario, and were up to 2.5*%* in Nova Scotia. In Yukon, the prevalence estimate given by all scenarios was 7.3*%*. Also, the TBI prevalence given by scenario 4 differed significantly across provinces/territories (*p*−*value*<0.0001)

MS had a prevalence estimate of around 2% in Nova Scotia and Ontario. There were no reported cases of MS among the HC clients in Yukon. There was no significant difference between the MS prevalence given by scenario 4 across provinces/territories (*p*−*value*=0.1).

### Prevalence estimates among persons receiving long-term care

Tables 4 – 5 show the six-month prevalence estimates and their associated exact 95% confidence intervals for all eight neurological conditions for long-term care residents by province/territory.

**Table 4:** Estimates of prevalence of neurological conditions by province/territory, among LTC home residents assessed between January 1st and July 1st, 2010

	**Province/Territory**											
**Condition**^***a***^	**British**		**Manitoba**		**Newfound. &**		**Nova Scotia**		**Ontario**		**Yukon**	
**&**	**Columbia**				**Labrador**							
**Scenario**^***b***^	**(%)**	**(CI)**^***c***^	**(%)**	**(CI)**	**(%)**	**(CI)**	**(%)**	**(CI)**	**(%)**	**(CI)**	**(%)**	**(CI)**
**ADRD**												
1	69.7	(69.0,70.4)	70.3	(69.1,71.5)	68.6	(63.7,73.2)	65.0	(61.1,68.7)	66.0	(65.7,66.4)	57.4	(48.4,66.0)
2	67.8	(67.1,68.6)	68.2	(67.0,69.4)	67.8	(62.9,72.5)	64.0	(60.1,67.8)	64.3	(64.0,64.6)	53.5	(44.5,62.3)
3	62.5	(61.7,63.2)	65.9	(64.6,67.1)	59.2	(54.0,64.1)	60.3	(56.4,64.2)	60.6	(60.2,60.9)	47.3	(38.4,56.3)
4 ^*d*^	58.9	(58.2,59.7)	62.6	(61.4,63.9)	56.3	(51.1,61.3)	60.3	(56.4,64.2)	57.9	(57.6,58.2)	41.9	(33.2,50.9)
5	34.8	(33.8,35.8)	43.9	(41.9,45.8)	28.9	(22.6,35.8)	NA ^*e*^		27.1	(26.8,27.5)	22.0	(14.6,31.0)
**PD**												
1	6.7	(6.4,7.1)	7.2	(6.5,7.9)	4.2	(2.4,6.7)	4.0	(2.6,5.8)	8.4	(8.2,8.6)	3.9	(1.3,8.8)
2	6.2	(5.8,6.6)	6.7	(6.0,7.3)	3.4	(1.8,5.7)	4.0	(2.6,5.8)	7.8	(7.6,8.0)	3.9	(1.3,8.8)
3	6.0	(5.6,6.4)	6.4	(5.8,7.1)	3.4	(1.8,5.7)	3.4	(2.1,5.1)	7.3	(7.2,7.5)	3.9	(1.3,8.8)
4	5.6	(5.2,5.9)	5.9	(5.3,6.5)	2.1	(0.9,4.1)	3.4	(2.1,5.1)	6.9	(6.8,7.1)	3.1	(0.9,7.7)
5	4.0	(3.6,4.4)	4.9	(4.1,5.8)	3.1	(1.1,6.6)	NA		3.6	(3.5,3.8)	2.8	(0.6,7.8)
**Epilepsy**												
1	4.0	(3.7,4.3)	4.7	(4.2,5.3)	7.3	(4.9,10.4)	3.2	(2.0,4.9)	6.3	(6.2,6.5)	7.8	(3.8,13.8)
2	3.7	(3.4,4.0)	4.7	(4.1,5.2)	6.8	(4.5,9.8)	3.0	(1.8,4.7)	6.0	(5.8,6.2)	6.2	(2.7,11.9)
3	3.5	(3.2,3.7)	4.4	(3.9,5.0)	6.8	(4.5,9.8)	2.7	(1.6,4.3)	5.5	(5.4,5.7)	6.2	(2.7,11.9)
4	3.2	(2.9,3.4)	4.2	(3.7,4.8)	6.5	(4.3,9.5)	2.7	(1.6,4.3)	5.2	(5.1,5.4)	5.4	(2.2,10.9)
5	1.6	(1.3,1.9)	1.0	(0.7,1.5)	2.6	(0.8,5.9)	NA		1.5	(1.4,1.6)	2.8	(0.6,7.8)
**TBI**												
1	3.4	(3.1,3.7)	1.6	(1.3,2.0)	2.4	(1.1,4.4)	0.6	(0.2,1.6)	4.0	(3.9,4.2)	3.9	(1.3,8.8)
2	2.5	(2.3,2.8)	1.1	(0.8,1.4)	1.8	(0.7,3.7)	0.5	(0.1,1.4)	2.5	(2.4,2.6)	3.9	(1.3,8.8)
3	2.4	(2.2,2.6)	1.0	(0.8,1.3)	1.6	(0.6,3.4)	0.3	(0.0,1.2)	1.9	(1.8,2.0)	3.1	(0.9,7.7)
4	1.4	(1.3,1.6)	0.6	(0.5,0.9)	1.6	(0.6,3.4)	0.3	(0.0,1.2)	0.9	(0.8,1.0)	1.6	(0.2,5.5)
5	2.2	(1.9,2.5)	1.0	(0.7,1.5)	1.5	(0.3,4.5)	NA		1.7	(1.6,1.8)	1.8	(0.2,6.5)

^*a*^ADRD = Alzheimer’s Disease and Related Dementias, PD = Parkinson’s Disease, TBI = Traumatic Brain Injury.

^*b*^See Table 1 for description of each of the five case definition scenarios.

^*c*^Exact 95% confidence interval.

^*d*^Only estimates by Scenario 4 are comparable across provinces/territories.

^*e*^No hospital or ED visits for LTC clients in this province.

**Table 5:** Estimates of the prevalence of neurological conditions by province/territory, among LTC home residents assessed between January 1st and July 1st, 2010

	**Province/Territory**											
**Condition**^***a***^	**British**		**Manitoba**		**Newfound. &**		**Nova Scotia**		**Ontario**		**Yukon**	
**&**	**Columbia**				**Labrador**							
**Scenario**^***b***^	**(%)**	**(CI)**^***c***^	**(%)**	**(CI)**	**(%)**	**(CI)**	**(%)**	**(CI)**	**(%)**	**(CI)**	**(%)**	**(CI)**
**MS**												
1	1.6	(1.4,1.8)	1.5	(1.2,1.9)	1.0	(0.3,2.7)	0.2	(0.0,0.9)	1.4	(1.4,1.5)	3.9	(1.3,8.8)
2	1.5	(1.3,1.7)	1.5	(1.2,1.9)	1.0	(0.3,2.7)	0.2	(0.0,0.9)	1.4	(1.3,1.5)	3.9	(1.3,8.8)
3	1.4	(1.2,1.6)	1.5	(1.2,1.8)	1.0	(0.3,2.7)	0.2	(0.0,0.9)	1.3	(1.3,1.4)	3.9	(1.3,8.8)
4^*d*^	1.4	(1.2,1.6)	1.5	(1.2,1.8)	0.8	(0.2,2.3)	0.2	(0.0,0.9)	1.3	(1.2,1.4)	3.9	(1.3,8.8)
5	0.8	(0.6,1.0)	1.5	(1.0,2.0)	1.0	(0.1,3.7)	NA ^*e*^		0.9	(0.8,0.9)	1.8	(0.2,6.5)
**CP**												
1	0.5	(0.4,0.6)	0.4	(0.3,0.6)	2.6	(1.3,4.8)	0.5	(0.1,1.4)	0.7	(0.6,0.7)	0.8	(0.0,4.2)
2	0.5	(0.4,0.6)	0.4	(0.3,0.6)	2.6	(1.3,4.8)	0.5	(0.1,1.4)	0.6	(0.6,0.7)	0.8	(0.0,4.2)
3	0.4	(0.3,0.5)	0.4	(0.2,0.5)	2.1	(0.9,4.1)	0.5	(0.1,1.4)	0.5	(0.5,0.6)	0.8	(0.0,4.2)
4	0.4	(0.3,0.5)	0.3	(0.2,0.5)	1.8	(0.7,3.7)	0.5	(0.1,1.4)	0.5	(0.5,0.6)	0.8	(0.0,4.2)
5	0.2	(0.2,0.4)	0.1	(0.0,0.3)	1.0	(0.1,3.7)	NA		0.2	(0.2,0.3)	0.0	(0.0,0.0)
**HD**												
1	0.3	(0.2,0.4)	0.3	(0.2,0.5)	0.5	(0.1,1.9)	0.2	(0.0,0.9)	0.3	(0.3,0.3)	0.8	(0.0,4.2)
2	0.3	(0.2,0.4)	0.3	(0.2,0.5)	0.5	(0.1,1.9)	0.2	(0.0,0.9)	0.3	(0.3,0.3)	0.8	(0.0,4.2)
3	0.3	(0.2,0.4)	0.3	(0.2,0.5)	0.5	(0.1,1.9)	0.2	(0.0,0.9)	0.3	(0.2,0.3)	0.8	(0.0,4.2)
4	0.3	(0.2,0.4)	0.3	(0.2,0.5)	0.5	(0.1,1.9)	0.2	(0.0,0.9)	0.3	(0.2,0.3)	0.0	(0.0,0.0)
5	0.2	(0.1,0.3)	0.2	(0.1,0.5)	0.5	(0.0,2.8)	NA		0.1	(0.1,0.2)	0.9	(0.0,5.0)
**ALS**												
1	0.5	(0.4,0.6)	0.1	(0.1,0.3)	0.3	(0.0,1.4)	0.3	(0.0,1.2)	0.3	(0.3,0.4)	0.0	(0.0,0.0)
2	0.4	(0.3,0.6)	0.1	(0.0,0.3)	0.3	(0.0,1.4)	0.3	(0.0,1.2)	0.3	(0.3,0.3)	0.0	(0.0,0.0)
3	0.4	(0.3,0.5)	0.1	(0.0,0.2)	0.0	(0.0,0.0)	0.2	(0.0,0.9)	0.2	(0.2,0.3)	0.0	(0.0,0.0)
4	0.3	(0.2,0.4)	0.1	(0.0,0.2)	0.0	(0.0,0.0)	0.2	(0.0,0.9)	0.2	(0.1,0.2)	0.0	(0.0,0.0)
5	0.3	(0.2,0.5)	0.1	(0.0,0.3)	0.0	(0.0,0.0)	NA		0.2	(0.1,0.2)	0.0	(0.0,0.0)

^*a*^MS = Multiple Sclerosis, CP = Cerebral Palsy, HD = Huntington’s Disease, ALS = Amyotrophic Lateral Sclerosis.

^*b*^See Table 1 for description of each of the five case definition scenarios.

^*c*^Exact 95% confidence interval.

^*d*^Only estimates by Scenario 4 are comparable across provinces/territories.

^*e*^No hospital or ED visits for LTC clients in this province.

Similar to the HC sector, ADRD was the neurological condition with the highest prevalence, although the estimates were much higher among persons in LTC homes. Based on scenario 1, where all sources of diagnostic information were used for case definition, the prevalence estimates ranged from 57.4*%* in Yukon to 70.3*%* in Manitoba.

The prevalence estimates given by scenario 4, where only the RAI 2.0 index assessment was used for case definition, ranged from 41.9*%* in Yukon to 62.6*%* in Manitoba. The *χ*^2^ test for homogeneity suggested that the prevalence of ADRD differed significantly across provinces/territories (*p*−*value*<0.0001).

The neurological condition with the second highest prevalence estimate among the LTC residents was PD. In Ontario, the prevalence ranged from 6.9*%* to 8.4*%* (excluding scenario 5). We see similar values in British Columbia and Manitoba. In Newfoundland and Labrador, Nova Scotia, and Yukon, the prevalence was less than 4%. The prevalence given by scenario 4 differed significantly across provinces/territories (*p*−*value*<0.0001).

The epilepsy prevalence estimate was as high as 7.8*%* in Yukon and 7.3*%* in Newfoundland and Labrador, as given by scenario 1. In Ontario, the prevalence ranged from 5.2*%* to 6.3*%*. The prevalence given by scenario 4 differed significantly across provinces/territories (*p*−*value*<0.0001).

The remaining neurological conditions had substantially lower prevalence estimates. TBI had a prevalence estimate ranging from 1% to 4% in Ontario, and from 0.3*%* to 0.6*%* in Nova Scotia.

MS had a prevalence estimate of less than 2% for all provinces, except for Yukon, where the estimate was 3.9*%* for all scenarios.

The prevalence estimate of CP was lower than 1% for all provinces/territories, except for Newfoundland and Labrador, where the estimates ranged from 1.8*%* to 2.6*%*.

The prevalence estimate of HD was less than 1% for all provinces/territories, whereas the prevalence for ALS was less than 0.5*%*.

### Prevalence estimates among persons receiving complex-continuing care

Table 6 shows the six-month prevalence estimates for all neurological conditions, for persons in CCC hospitals/units in Ontario and Manitoba.

**Table 6:** Estimates of the prevalence of neurological conditions by province/territory, among Complex Continuing Care (CCC) hospital patients assessed between January 1st and July 1st, 2010

**Province &**	**Condition**^***a***^							
**Scenario**^***b***^	**ADRD**	**PD**	**EPILEPSY**	**TBI**	**MS**	**CP**	**HD**	**ALS**
**Ontario**								
1	33.6^*c*^ (32.7,34.5)^*d*^	6.4 (5.9,6.9)	8.0 (7.5,8.5)	5.6 (5.2,6.1)	2.4 (2.2,2.8)	1.1 (0.9,1.3)	0.4 (0.3,0.5)	1.1 (0.9,1.3)
2	32.6 (31.7,33.6)	6.1 (5.6,6.6)	7.3 (6.8,7.8)	4.6 (4.2,5.0)	2.3 (2.0,2.6)	0.9 (0.7,1.1)	0.3 (0.2,0.5)	0.9 (0.7,1.1)
3	29.7 (28.9,30.6)	5.7 (5.3,6.1)	6.7 (6.3,7.2)	4.0 (3.6,4.4)	2.2 (1.9,2.5)	0.8 (0.6,1.0)	0.3 (0.2,0.5)	0.8 (0.6,1.0)
4	26.6 (25.8,27.5)	5.0 (4.6,5.5)	6.1 (5.7,6.6)	2.5 (2.2,2.8)	2.1 (1.8,2.4)	0.7 (0.5,0.9)	0.3 (0.2,0.4)	0.6 (0.5,0.8)
5	15.9 (15.2,16.7)	4.0 (3.6,4.4)	2.3 (2.0,2.6)	2.4 (2.1,2.8)	1.4 (1.2,1.7)	0.5 (0.3,0.6)	0.2 (0.1,0.3)	0.5 (0.4,0.7)
**Manitoba**								
1	21.7 (15.2,29.6)	5.8 (2.5,11.1)	9.4 (5.1,15.6)	11.6 (6.8,18.1)	5.8 (2.5,11.1)	0.7 (0.0,4.0)	0.7 (0.0,4.0)	1.4 (0.2,5.1)
2	20.3 (13.9,28.0)	5.1 (2.1,10.2)	8.0 (4.0,13.8)	11.6 (6.8,18.1)	5.8 (2.5,11.1)	0.7 (0.0,4.0)	0.7 (0.0,4.0)	0.7 (0.0,4.0)
3	18.8 (12.7,26.4)	5.1 (2.1,10.2)	7.2 (3.5,12.9)	11.6 (6.8,18.1)	4.3 (1.6,9.2)	0.7 (0.0,4.0)	0.7 (0.0,4.0)	0.7 (0.0,4.0)
4	15.9 (10.3,23.1)	5.1 (2.1,10.2)	7.2 (3.5,12.9)	6.5 (3.0,12.0)	4.3 (1.6,9.2)	0.7 (0.0,4.0)	0.7 (0.0,4.0)	0.7 (0.0,4.0)
5	15.8 (9.3,24.4)	3.0 (0.6,8.4)	0.0 (0.0,0.0)	7.9 (3.5,15.0)	3.0 (0.6,8.4)	1.0 (0.0,5.4)	0.0 (0.0,0.0)	1.0 (0.0,5.4)

^*a*^ADRD = Alzheimer’s Disease and Related Dementias, PD = Parkinson’s Disease, TBI = Traumatic Brain Injury, MS = Multiple Sclerosis, CP = Cerebral Palsy, HD = Huntington’s Disease, ALS = Amyotrophic Lateral Sclerosis.

^*b*^See Table 1 for description of each of the five case definition scenarios.

^*c*^Prevalence estimate expressed as a percentage (%).

^*d*^Exact 95% CI.

Again, ADRD was the condition with the highest prevalence estimates, although the estimates were not as high as in the LTC sector. In Ontario, the prevalence of ADRD ranged from 26.6*%* to 33.6*%*, whereas in Manitoba it ranged from 15.9*%* to 21.7*%*.

In Ontario, the prevalence estimates for epilepsy ranged from 6.1*%* to 8% and were higher than in the LTC sector. For PD, the prevalence estimates were about 2% lower in CCC than in LTC in Ontario, with estimated ranging from 5% to 6.4*%*.

Also, slightly higher prevalence estimates were seen in the CCC sector in Ontario for TBI, MS, and ALS, when compared to the ones among LTC residents.

### Prevalence estimates among persons receiving mental-health care – Ontario

Table 7 shows the six-month prevalence estimates for seven neurological conditions, for the Ontario MH cohort. The prevalence estimate for ALS ranged from 0.02*%* to 0.03*%*.

**Table 7:** Estimates of the prevalence of neurological conditions among Ontario Psychiatric Hospital Inpatients assessed between January 1st and July 1st, 2010

	**Condition**^***a***^						
**Scenario**^***b***^	**ADRD**	**PD**	**Epilepsy**	**TBI**	**MS**	**CP**	**HD**
1	10.0 ^*c*^ (9.6,10.4) ^*d*^	1.7 (1.5,1.8)	3.0 (2.7,3.2)	2.6 (2.4,2.8)	0.3 (0.3,0.4)	0.3 (0.3,0.4)	0.3 (0.2,0.3)
2	9.0 (8.7,9.4)	1.3 (1.1,1.4)	2.4 (2.2,2.6)	1.8 (1.6,2.0)	0.3 (0.2,0.4)	0.3 (0.3,0.4)	0.3 (0.2,0.3)
3	6.9 (6.6,7.3)	1.0 (0.9,1.2)	1.9 (1.8,2.1)	1.5 (1.3,1.6)	0.3 (0.2,0.3)	0.3 (0.2,0.3)	0.2 (0.2,0.3)
4	6.3 (6.0,6.6)	0.5 (0.4,0.6)	0.6 (0.5,0.7)	0.1 (0.1,0.2)	0.1 (0.1,0.2)	0.2 (0.1,0.2)	0.2 (0.1,0.3)
5	3.3 (3.0,3.5)	0.8 (0.7,0.9)	1.6 (1.5,1.8)	1.5 (1.3,1.6)	0.2 (0.2,0.3)	0.2 (0.1,0.3)	0.2 (0.1,0.2)

^*a*^ADRD = Alzheimer’s Disease and Related Dementias, PD = Parkinson’s Disease, TBI = Traumatic Brain Injury, MS = Multiple Sclerosis, CP = Cerebral Palsy, HD = Huntington’s Disease.

^*b*^See Table 1 for description of each of the five case definition scenarios.

^*c*^Prevalence estimate expressed as a percentage (%).

^*d*^Exact 95% CI.

Among the persons in inpatient psychiatry, the ADRD prevalence estimates ranged from 6.3*%* to 10%. Epilepsy and TBI had prevalence estimates up to 3%, whereas the PD prevalence estimates were less than 2%.

CP, HD, and MS had prevalence estimates lower than 0.5*%*.

## Discussion

This study provided new information regarding the prevalence of eight neurological conditions within four clinical settings in the continuum of care that had received little attention in the literature. For each condition, cases were identified using data from the interRAI assessments and other databases such as acute and ambulatory care, using data linkage. The study provided lower and upper bounds for the estimates of a six-month period prevalence using five different case definitions. These scenarios used different combinations of diagnostic sources and different lengths of look-back windows for historical data. This was the first Canadian or international study to our knowledge to provide prevalence estimates within all four care settings. In most sectors, the study results were based on full-population data at the provincial/territorial level from January through June 2010, except for Newfoundland and Labrador and Nova Scotia for the LTC sector, and Manitoba for the CCC sector. Results for persons in LTC facilities from Manitoba were based on full-population data at a regional level (WRHA).

The neurological condition with the highest prevalence estimate in all four care settings was ADRD, which probably reflects the advanced age and functional impairments typical of persons in these settings. In the HC sector, the upper bound of the ADRD prevalence estimate was around 25% in Nova Scotia and Ontario, and around 9% in Yukon. The upper bound of the estimate ranged from 57% to 70% among persons receiving LTC in six provinces/territories, and was as high as 34% among CCC patients in Ontario. The LTC results are comparable to previous studies [4,24,25]. Also, 10% of inpatient mentalhealth patients in Ontario were identified as ADRD cases when all sources of diagnostic information are considered.

The prevalence of ADRD and other neurological conditions in home care and long-term care varied across regions. For example, persons receiving care in the Yukon often had notably different estimated prevalence compared with other regions. Future research is needed to understand whether these regional variations reflect differences in policy or practice across provinces or differences in the characteristics of the underlying populations.

The prevalence estimate of PD was as high as 8% in the LTC sector, which is reasonably comparable to the estimate provided by a previous Canadian study (Moghal et al. [8] reported a prevalence estimate of 6% among nursing home residents). The PD prevalence was around 5% among persons receiving HC in Nova Scotia and Ontario, and was around 6% among Ontario complex continuing care hospital patients. The upper bound of the PD prevalence estimate was around 2% in the Ontario psychiatric hospital sector.

The prevalence estimate for epilepsy was as high as 8% in the LTC and CCC sectors, and was around 3% among mental health patients in Ontario. These estimates are higher than those seen in the general population (e.g., Tellez-Zenteno et al. [5] estimated the prevalence to be around 0.5−0.6*%*). However, this is not unexpected considering that the prevalence of epilepsy increases with age and the individuals studied here were expected to be less healthy than the general population, with more comorbid conditions that can be associated with epilepsy (e.g., stroke, neurodegenerative conditions).

Among persons receiving LTC in Ontario and Yukon, 4% were identified as TBI cases when all diagnostic sources were used. In the home-care sector in Ontario and Nova Scotia, and in the mental-health care sector in Ontario, the prevalence estimate for TBI was around 3%, whereas in the CCC sector in Ontario it was 6%. Prevalence studies of TBI are extremely rare, as most studies focus on the incidence of TBI. One Canadian study reported an age-standardized prevalence of TBI of 50.4 per 100,000 in 2001-2002 [26].

The upper bound for the prevalence estimate of MS was less than 2% among LTC clients in all five provinces, and among HC clients in Ontario and Nova Scotia, and it was 4% among LTC clients in Yukon. Similarly to epilepsy, these estimates were higher than those reported in the general Canadian population [6], ranging from 56 to 298 per 100,000, possibly reflecting the complex physical, cognitive, and psychosocial demands of this progressively disabling disease. Among people over 65 years with MS living in Canada, Finlayson [27] found nursing home estimates of 5.8*%* (of 274) and 9.2*%* (of 142), similar to those reported in the United States [28,29].

For ALS, CP, and HD, the prevalence estimates were lower than 1% in the LTC and HC sectors, except for the prevalence of CP in LTC in Newfoundland and Labrador, where the estimate was 3%. In Ontario, the prevalence estimates for these conditions were slightly higher in the CCC sector than in the LTC sector. However, the prevalence of ALS and HD were about 100 fold higher in these settings than what has been reported in the general population, at 4.9 and 2.71 per 100,000 respectively [12,30]. This is not surprising as patients with ALS and HD would be expected to be primarily cared for in these settings, due to the significant functional deficits they experience [31,32].

Previous research has provided evidence of the validity of diagnostic information in these various interRAI databases [33]. The interRAI assessment approach requires the use of all sources of information as evidence to be considered by assessors, including medical records, interviews, and observations of the person being assessed, and consultation with family members and other health professionals. Although this approach is the standard across all provinces/territories using the instruments in Canada, it may be the case that variations in access to specialized diagnostic services may account for some of the regional variations in prevalence estimates. For example, differences in the availability of advanced medical testing (e.g., access to genetic testing or clinical evaluation by neurologists or geriatricians) may affect the ascertainment of cases from the medical record thereby influencing prevalence estimates.

It might be the case that eligibility criteria could affect the prevalence estimates within specific sectors, but it is unlikely that this is an explanation for the differences between the Canadian provinces/territories reported here. Studies comparing the nursing home [19] and home care [34] populations across Canada showed only modest differences in the case mix distributions and need profiles of persons in these care settings in different provinces/territories.

The results demonstrated that neurological conditions are an important source of morbidity in complex continuing care hospital, nursing home, and home care settings. These prevalence estimates were considerably higher than estimates based on the general population as a whole [5,6]. The high prevalence estimates, particularly for conditions like dementia and Parkinson’s Disease, suggest that attention must be paid to ensure that staff in those settings have appropriate training and expertise to deal with the needs of persons with these conditions. The extent to which the policies and practices in different care settings support the needs of people with neurological conditions requires urgent examination.

Although the neurological conditions considered here are much less common in inpatient psychiatry, they were present, and highlighted the well-recognized burden of psychiatric comorbidity in neurologic diseases [35-37]. Persons with comorbid neurological conditions in psychiatry may have special needs not evident in the general population served by mental health care providers. Multiple sclerosis is associated with a very high prevalence of mood disorders in the general population [36], yet the prevalence estimates of MS in the mental health settings were only slightly higher than that of the general population. A possible explanation is that psychiatric hospital settings may not be viewed as a suitable care setting for people with MS due to their medical needs.

From a methodological perspective, the range of prevalence estimates was not greatly affected by the case definition assumptions made in the different case definition scenarios. The only clear outliers in prevalence estimates came from the exclusive use of DAD and NACRS records which almost certainly yield substantial under-estimates of the true prevalence of these conditions. Although all available records in the DAD and NACRS diagnosis section were used for case definition of neurological conditions (i.e., “most responsible diagnosis” and other diagnoses), there may have been cases where the neurological conditions of interest were not recorded at all because they were not the main reason for being admitted. It is well known that comorbid diagnoses are less likely to be included in the hospital or ER discharge records [38,39]. Therefore, it is not surprising that some cases are missed when the case definition is based on the DAD and NACRS records only, which results in under-estimation of the prevalence.

Therefore, the interRAI instruments are more useful for obtaining prevalence estimates across the continuum of care than acute hospital-based administrative records. However, as clinical assessment instruments the interRAI instruments also have great value because they provide a broad range of clinical indicators that can be used to further examine the clinical characteristics of persons with neurological conditions. Future efforts to refine HCRS, CCRS, and OMHRS should explore options to further enhance the diagnostic items in the interRAI assessments to conveniently (and reliably) capture diagnostic data not currently on the standard pick list.

The focus of this study was to explore different methodologies for comparing the period prevalence over a certain time interval (i.e., January through June, 2010). In the future, it would interesting to investigate how the prevalence in the four sectors of interest changes over time, and to compare the trends for the prevalence as defined by different scenarios and across provinces. In addition, the present study focused on prevalence estimates within a sector in a given time period; however, individuals were not constrained to appearing in only a single sector. Therefore, it may be the case that persons who were admitted from home care to nursing homes would contribute to the period prevalence estimates of both care settings by virtue of having received services and assessments in both settings. This may be problematic if one were estimating prevalences in the total population based on these data, but it is not a concern when estimating prevalences within individual sectors.

## Limitations

This study had several limitations. First, due to gradual implementation of the interRAI instruments, some provinces had more historical interRAI data than others, which makes the provincial prevalence estimates given by some scenarios not comparable. This issue will become less important over time as these provinces continue to widely implement these instruments. Second, not all provinces/territories reported acute or ambulatory care abstracts (e.g., Yukon). Therefore, comparisons between provinces are not recommended when the case definition involves historical data from the DAD and/or NACRS. Also, some Canadian provinces/territories have just recently started implementing interRAI instruments (e.g., Alberta), whereas others have not implemented any of them (e.g., Quebec). Therefore, national-level prevalence estimates for neurological conditions within all four care settings are not possible at this time. An alternative to the approach taken here, where partial data were used for some provinces/territories, would have been to consider only provinces/territories with complete interRAI data (e.g., Ontario for the LTC and HC sectors). However, this approach would have drastically limited the analysis and would have omitted useful information.

The RAI-HC diagnoses pick list includes only four neurological conditions (i.e., ADRD, PD, MS, and TBI) and the ICD-10-CA codes were not available for the other conditions. Therefore, for the HC sector, prevalence estimates could not be provided for some of the common neurological conditions such as epilepsy. In addition, the older versions of interRAI instruments employed here have some variability in diagnostic terminology (e.g., some refer to parkinsonism and other Parkinson’s disease); however, these variations have been eliminated in newer versions of these instruments to adhere to a single cross-sector standard [13].

In the RAI-MH instrument, dementia can be captured in three ways: by recording the corresponding ICD-10-CA codes, by checking the item “delirium, dementia and amnesic and other cognitive disorders” in the provisional diagnostic section, or by entering the DSM-IV codes, in the psychiatric diagnosis section. However, in some cases the provisional diagnosis item “delirium, dementia and amnesic and other cognitive disorders” may be checked off to identify a diagnosis other than dementia, which might inflate the prevalence estimate of dementia.

As part of the on-going research related to the development of interRAI instrument, studies of the validity of the diagnostic codes on these assessments have been done in the nursing home and CCC sectors compared with acute hospital records [40,41]. Forthcoming research arising from the ideasPNC project extends this research to include the HC and MH sectors (results available on request). Clinicians completing interRAI assessments are trained to use all sources of information available including direct observation of the person, interviews with other informants, consultations with other colleagues, medical records, and the chart. They then use their clinical judgement to record observations on all interRAI items, including diagnostic codes. Nonetheless, the potential for misdiagnosis, under-ascertainment or over-ascertainment of certain conditions remains. It would be helpful for further research to be undertaken comparing the interRAI assessment data against systematic, expert reviews of longitudinal medical records or direct patient assessment by neurologists to more fully evaluate the validity of those items. Further, ICD coding, and in particular ICD-10 coding has not been validated for most neurological conditions. Some codes are highly specific such as MS which has a single code, while others are nonspecific [42].

Two other data sources have been used in previous studies for case identification [43]. These include physician services/billing and prescription medication data. These additional sources of information might help identify even more cases of persons with neurological conditions across the continuum of care, and should be considered in future prevalence studies.

## Conclusion

Compared with previous reports on the prevalence of neurological conditions in the general population, there is a substantially higher proportion of Canadians in non-acute health care settings affected by these conditions. This was true for the most common conditions like Alzheimer’s and related dementias, but it also applied to less prevalent neurological conditions. As a result, it is particularly important for staff to have the expertise and resources to respond effectively to the strengths, preferences, and needs of this population. While hospital and emergency department records provided useful supplementary information for estimating the prevalence of various neurological conditions, they would substantially underestimate the true prevalence of these conditions if used on their own. However, interRAI assessment records provide a helpful source of information for obtaining these estimates in nursing home, home care, and mental health settings. These assessments can provide evidence about the prevalence of neurological conditions as well as important multidimensional evidence on the clinical characteristics and service utilization patterns of this growing population.

## Abbreviations

ADRD: Alzheimer’s disease and related Dementia; ALS: Amyotrophic lateral sclerosis; CP: Cerebral palsy; CCRS: Continuing care reporting system; CIHI: Canadian Institute for Health Information; DAD: Discharge abstract database; ED: Emergency department; HC: Home care; HCRS: Home care reporting system; HD: Huntington’s disease; ICD-10-CA: International classification of diseases, version 10 Canada; ideasPNC: Innovations in data, evidence and applications for persons with neurological conditions; LTC: Long-term care; CCC: Complex-continuing care; RAI 2.0: Resident assessment instrument –minimum data set 2.0; MH: Mental health; MS: Multiple sclerosis; NACRS: National ambulatory care reporting system; OMHRS: Ontario mental health reporting system; PD: Parkinson’s disease; RAI-HC: Resident assessment instrument – Home care; RAI-MH: Resident assessment instrument – Mental health; TBI: Traumatic brain injury.

## Competing interests

The authors declare that they have no competing interests.

## Authors’ contributions

JP Hirdes, CJ Maxwell, and N Jetté conceived of the study. O Danila performed the statistical analyses and drafted the manuscript. All authors participated in the study design, revised and approved the final manuscript.

## Pre-publication history

The pre-publication history for this paper can be accessed here:

http://www.biomedcentral.com/1472-6963/14/29/prepub

## Supplementary Material

Additional file 1 This file includes Tables S1–S5 that contain details about the data sets used in the study, and Tables S6–S8 that contain details about the interRAI diagnostic items and ICD-10-CA codes used for case definitions. Click here for file
