# Supplementary material for: Prevalence of neurological conditions across the continuum of care based on interRAI assessments
Source: BMC Health Serv Res. 2014 Jan 22;14:29. doi: 10.1186/1472-6963-14-29 (PMC3906754; doi:10.1186/1472-6963-14-29)
Supplement: Additional file 1 — This file includes Tables S1–S5 that contain details about the data sets used in the study, and Tables S6–S8 that contain details about the interRAI diagnostic items and ICD-10-CA codes used for case definitions. [file 1472-6963-14-29-S1.pdf]

Table S1: Number of Home Care Clients Assessed with the RAI-HC by Year and Province/Territory

Year	Nova Scotia	Ontario	Yukon
2001	1,091	0	0
2002	3,929	0	0
2003	9,126	15	0
2004	11,060	21	0
2005	11,675	48	0
2006	11,854	205	28
2007	12,563	102,632	79
2008	12,477	126,462	102
2009	13,241	135,378	141
2010	4,583	132,626	161

Table S2: Number of Persons in Long-Term and Complex-Continuing Care Assessed with the RAI 2.0 by Year and Province/Territory

Year	British Columbia	Manitoba	Newfound.& Labrador	Nova Scotia	Ontario	Yukon
2003	1	0	0	3	14,117	0
2004	0	0	0	348	18,303	0
2005	0	0	0	483	21,254	0
2006	184	0	0	470	32,263	0
2007	1,843	15	0	511	41,591	0
2008	3,997	4,580	37	508	52,688	148
2009	18,409	6,655	49	716	95,593	143
2010	19,163	6,721	497	752	107,270	153

Table S3: Number of Mental Health Inpatients Assessed with the RAI-MH in Ontario by Year of Assessment

Year	No. Persons
2005	8,211
2006	37,428
2007	38,875
2008	38,550
2009	38,621
2010	31,195

Table S4: Number of Persons with at Least One interRAI Assessment and Hospital Visit by Year and Province

Year	Alberta	British Columbia	Manitoba	New Brunswick	Newfound.& Labrador	Nova Scotia	Ontario	Prince Edward
2001	0	0	4	0	0	4	54	0
2002	0	1	5	0	0	5	85	0
2003	1	13	19	0	2	61	364	0
2004	46	1,808	729	149	49	8,870	48,477	15
2005	88	3,324	1,111	187	87	10,771	83,851	25
2006	156	5,032	1,675	231	125	10,697	99,970	32
2007	177	6,969	2,269	231	218	10,753	118,793	43
2008	180	8,383	2,787	234	269	10,619	129,810	44
2009	158	9,384	3,182	175	337	8,912	130,277	43
2010	109	8,178	2,650	73	306	4,804	122,159	34

Table S5: Number of Persons with at Least One interRAI Assessment and Emergency Department Visit by Year and Province/territory

Year	British Columbia	Nova Scotia	Ontario	Prince Edward
2002	13	0	24,212	0
2003	54	564	57,458	6
2004	101	1,300	101,842	10
2005	146	2,109	160,622	18
2006	240	1,661	218,062	21
2007	655	1,561	259,950	21
2008	461	1,492	273,738	28
2009	257	1,206	259,418	22
2010	216	732	224,144	14

Table S6: RAI-HC Diagnoses Information – Section J

**Neurological Condition** [a]	**Item** [b]
Alzheimer's Disease and Related Dementias	j1g, j1h
Multiple Sclerosis	j1k
Parkinsonism	j1l
Head Trauma	j1i

[a]Disease is recorded if present and monitored or treated by home care professional or present and not subject to treatment or monitoring

[b]Items j2a – j2d correspond to ICD-10-CA Codes for "Other current diagnosis". HCRS data set does not include these items.

Table S7: RAI 2.0 Disease Diagnoses – Section I

**Neurological Condition** [a]	**Item** [b]
Alzheimer's Disease and Related Dementias	i1r, i1v
Amyotrophic Lateral Sclerosis	i1q
Cerebral Palsy	i1t
Seizure Disorder	i1cc
Huntington's Chorea	i1x
Multiple Sclerosis	i1y
Parkinson's Disease	i1aa
Traumatic Brain Injury	i1ee

[a]Disease is recorded if it has a relationship to current ADL, cognitive, mood, and behaviour status, medical treatments, nurse monitoring, or risk of death

[b]Items i3a – i3f correspond to ICD-10-CA Codes for "Other current diagnosis"

Table S8: ICD-10-CA codes for Neurological Conditions

**Neurological Condition**	**ICD-10-CA** [a]
Alzheimer's Disease and Related Dementias	G30, F00, F01, F02, F03
Cerebral Palsy	G80
Epilepsy	G40
Huntington's Disease	G10, F02.2
Motor Neuron Disease	G12
Multiple Sclerosis	G35
Parkinsonism	F02.3, G20, G21, G22
Traumatic Brain Injuries	F07.2, S02.0, S02.1, S02.3, S02.7, S02.8, S02.9, S06, S07,T06.0, T90.5

[a]These are the codes used for neurological conditions for all care settings
